# Retraction: The contribution of N_2_O_3_ to the cytotoxicity of the nitric oxide donor DETA/NO: an emerging role for S-nitrosylation

**DOI:** 10.1042/BSR20120120_RET

**Published:** 2026-02-04

**Authors:** Ahlam A. Ali, Jonathan A. Coulter, Claire H. Ogle, Marie M. Migaud, David G. Hirst, Tracy Robson, Helen McCarthy

**Keywords:** cytotoxicity, hypoxia, nitric oxide, S-nitrosylation

This article is being retracted from *Bioscience Reports* at the request of the authors following receipt of a notification from a reader alerting the Editorial Office to multiple similarities that exist both within the manuscript and between this article and others by overlapping authors. The specific similarities are listed below:

Similarities between this article and papers published elsewhere:

- Figure S5 DU145/H2B (21% O_2_) and the last 4 bands of Figure 5 GAPDH (flipped and compressed) from DOI: 10.1016/j.ejpb.2018.02.029
- Figure S4 DU145/B-actin (21% O_2_) and the first 4 bands of Figure 5B GAPDH from DOI: 10.1016/j.nano.2016.11.019
- Figure 8A MDA-MB-231 (first 7) bands and the Figure 5A MV4-11 (39KDa) bands from DOI: 10.3390/ijms24065717
- Figure 8A B-actin (first 7) bands and the Figure 5A Kasumi-1/GAPDH bands (flipped) from DOI: 10.3390/ijms24065717
- Figure S4 DU145/PARP bands (first 3 columns) and the Figure 3Bn MV4-11/PARP (last 3 columns) bands from DOI: 10.3390/ijms24065717

Similarities within the article:

- Figure S4 MDA-MB-231/C-Caspase 9 (21% O_2_) with Figure S5 MDA-MB-231/SNO GAPDH (21% O_2_) bands
- Figure S4 DU145/Total caspase 3 (0.1% O_2_, flipped) with Figure S5 DU145/SNO GAPDH (21% O_2_) bands
- Figure S5 DU145/SNO HIF-1a 8h, 18h and 24h (21% O_2_) bands with the DU145/SNO p53 Con, 8h and 18h (0.1% O_2_) bands.
- Figure S4 L132/Total caspase 3 (21% O_2_) and the Figure S5 DU145/Total HIF-1a (0.1% O_2_) bands
- Figure S4 MDA-MB231/C-caspase-3 (0.1% O_2_) and the Figure S5 L132/SNO GAPDH (21% O_2_) bands
- Figure S5 MDA-MB231/Total p53 (21% O_2_) and the L132/SNO GAPDH (0.1% O_2_) bands
- Figure S5 DU145/SNO p53 8h, 18h and 24h (21% O_2_) and the L132/SNO p53 Con, 8h and 18h (21% O_2_) bands.

The authors cooperated with the investigation but are unable to correct the article. Confidence in the data published as well as those provided during the investigation has been lost by both the authors and the Editorial Board. The authors therefore wish to retract the article; the Editor-in-Chief and Editorial Board agree with the retraction.

